# Editorial

**Published:** 1864-05

**Authors:** 


### Editorial.

### A CHLOROFORM CASE.

The following was related to us by Dr. Benedict of Detroit. On the 9th of January last, a young woman, of rather full habit, sanguine temperament, and of apparently good health, applied to have a molar tooth extracted; she desired chloroform. The doctor thought he saw in her appearance some counter-indication, but made an application of chloroform locally, and gave a very few inhalations, not enough to produce anæsthesia, and removed the tooth. There was no unusual hæmorrhage, or unfavorable results of any kind.

On Feb. 16th following, the woman called upon Dr. M. a neighboring Dentist for the removal of a bicuspid tooth; she this time also desired chloroform, which was given her, producing complete anæsthesia. She recovered from the influence slowly, and had unpleasant sensations afterwards, accompanied with dizziness, fullness of the head, and feebleness of the lower extremities. On the morning of Saturday the 19th, about 9 o'clock, excessive hæmorrhage set in. Dr. M. was called in, and applied a loose pledget of cotton, with a piece of cork placed upon it, then bandaged the jaws to make compression. The hæmorrhage continued unabated. A student of homœopathy was called, but did nothing. Things continued thus till Monday the 21st, 2 o'clock in the afternoon, when Dr. B. was sent for. He found the patient in rather a bad condition, the blood flowing freely, the mouth full of decomposing and offensive clots of blood, and very sore, the face flushed, the head hot and painful, with evidently a strong determination of blood to the head, the extremities cold, the pulse about 130 per minute.

The mouth was cleansed out as thoroughly as possible, and a piece of sponge, rolled tightly, and conforming to the cavity as nearly as possible, and of a size to be introduced readily, was forced in; this stopped the flow of blood at once, which did not return.

The patient was put upon general treatment, cold applied to the head, and warmth to the extremeties. The patient very soon began to improve, pulse diminished rapidly, the pain in the head was relieved. At this point the case was put into the hands of a physician who gave proper treatment, and in about one week she recovered so as to resume her usual avocation.

This is rather an interesting case, though perhaps it should have been more minutely given. The extent of the chloroform influence in it, may be a debatable question, though it seems very much as though the chloroform induced great determination of blood to the head, and if so, the train of conditions that followed. Is chloroform liable to produce determination of blood to the head or to any part. T.

### SUGGESTIONS TO THE DENTIST'S ASSISTANT.

But few dentists avail themselves of the advantages derivable from an assistant. Perhaps none avail themselves of an assistant at the chair except those who use the mallet, and none so much require it. It has been suggested, that the employment of an assistant involves expense, true, so does eating one's dinner. But if operations can be more rapidly or better performed by the aid of an assistant than without, then certainly the expense is not to be considered.

It has also been suggested that the presence of a third person is objectionable; that of course will be modified by the character of the assistant; no reasonable person would object to the presence of an assistant when he or she was to derive material advantage therefrom.

Several things will modify the efficiency of an assistant, in the use of the mallet especially; he should be able to fix, and keep his attention upon the operations, and have a considerable knowledge of the various parts of an operation; he should always feel an interest in every operation at which he assists, should be able to make uniform and well regulated time strokes, should also be quick to make indicated movements, and be able to pass from one to the other with facility.

An attentive assistant can do much in other particulars that will greatly facilitate the operator, such as, preparing the gold, keeping in place instruments, appliances, &c. No operator who has had a good assistant, till he has becomes familiar with the advantages derivable therefrom, would think of being without.

A good assistant should possess ability little inferior to that of the operator himself. T.

### THE PEOPLE'S DENTAL JOURNAL.

The April number of this excellent work has just come to hand. We think it better than its predecessors, and it can well afford to be, for it now has three editors instead of one as before; and though they all contributed to its pages before, they of course will now feel a warmer interest in its success. If one editor could put the journal on a permanent basis, certainly three can keep it there. The People's Dental Journal, we consider as devoted to one of the most important points connected with our profession, and will give it our feeble aid, and bid it all speed.

We would suggest that every local society should engage in the work to which the Journal is devoted, either by availing themselves of the advantages of this one or some other, or each one publishing for their own use—what seems to them best adapted to their locality: such a course however would perhaps not be the most economical. Efficiency however should be the great point.

Educate the people by some means, and all means. T.

### AMERICAN DENTAL ASSOCIATION.

The fourth Annual Meeting of the American Dental Association will be held at Niagara Falls, the last Tuesday in July next, at ten o'clock A. M.

It is hoped that all members will manifest an active interest by being present. CHARLES R. BUTLER, *Sec'y.*

